# Impact of antibiotic usage on extended-spectrum β-lactamase producing *Escherichia coli* prevalence

**DOI:** 10.1038/s41598-021-91332-x

**Published:** 2021-06-22

**Authors:** Jeong Yeon Kim, Yunjin Yum, Hyung Joon Joo, Hyonggin An, Young Kyung Yoon, Jong Hun Kim, Jang Wook Sohn

**Affiliations:** 1 Division of Infectious Diseases, Department of Internal Medicine, Korea University College of Medicine, Seoul, Republic of Korea; 2 Department of Biostatistics, Korea University College of Medicine, Seoul, Republic of Korea; 3 Korea University Research Institute for Medical Bigdata Science, Korea University, Seoul, Republic of Korea; 4 Department of Cardiology, Cardiovascular Center, Korea University College of Medicine, Seoul, Republic of Korea; 5 Division of Infectious Diseases, Department of Internal Medicine, CHA Bundang Medical Center, CHA University, Seongnam, Republic of Korea

**Keywords:** Infection, Computational biology and bioinformatics

## Abstract

An increase in antibiotic usage is considered to contribute to the emergence of antimicrobial resistance. Although experts are counting on the antimicrobial stewardship programs to reduce antibiotic usage, their effect remains uncertain. In this study, we aimed to evaluate the impact of antibiotic usage and forecast the prevalence of hospital-acquired extended spectrum β-lactamase (ESBL)—producing *Escherichia coli* (*E. coli*) using time-series analysis. Antimicrobial culture information of *E. coli* was obtained using a text processing technique that helped extract free-text electronic health records from standardized data. The antimicrobial use density (AUD) of antibiotics of interest was used to estimate the quarterly antibiotic usage. Transfer function model was applied to forecast relationship between antibiotic usage and ESBL-producing *E. coli*. Of the 1938 hospital-acquired isolates, 831 isolates (42.9%) were ESBL-producing *E. coli*. Both the proportion of ESBL-producing *E.* coli and AUD increased over time. The transfer model predicted that ciprofloxacin AUD is related to the proportion of ESBL-producing *E. coli* two quarters later. In conclusion, excessive use of antibiotics was shown to affect the prevalence of resistant organisms in the future. Therefore, the control of antibiotics with antimicrobial stewardship programs should be considered to restrict antimicrobial resistance.

## Introduction

Antimicrobial resistant bacteria are considered as a significant cause of mortality and socioeconomic burden globally. Of all resistant organisms, extended-spectrum β-lactamase (ESBL)-producing gram-negative bacteria are characterized as the most critical and top priority antimicrobial-resistant pathogens by the World Health Organization (WHO)^1^. Several studies on risk factors of ESBL-producing organism infections indicate that it is strongly associated with prior usage of antibiotics.

Various classes of antibiotics have been described as risk factors for the prevalence of ESBL-producing organisms, including cephalosporins, carbapenems, and trimethoprim/sulfamethoxazole^2,3^. These previous studies were based on the concept that using antimicrobial drug results in selective pressure toward the emergence of resistance^4^. However, the direct consequence of specific antibiotic usage on antimicrobial resistance has not been established yet^5,6^.

An antibiotic or antimicrobial stewardship program is a coordinated intervention established by healthcare professionals to select the most appropriate antibiotic, duration, dose, and route of administration for a given patient with an intention to minimize the impact on antibiotic resistance^7^. Most tertiary hospitals are implementing antibiotic stewardship programs even if there is insufficient evidence regarding any relationship between selective antibiotic consumption and antimicrobial resistance. There have been only a few attempts to assess the impact of antibiotic stewardship program using the in-hospital data^8,9^.

One of the challenges in elucidating this relationship is that the process of analyzing the antimicrobial data and measurement of the level of prior usage of antibiotic is complicated^10^. The evaluation of time-dependent variables requires the establishment of a complex analysis system^11^. However, the implementation of big data analysis models on hospital electronic health record (EHR) systems made this easier. The development of a test-processing algorithm that can convert text-wise EHR data to standardized data has made it possible to process the immense data, including antimicrobial resistance phenotypes.

In the present study, we aimed to investigate the relationship between the volume of antibiotic consumption and proportion of hospital-acquired ESBL-producing *Escherichia coli* (*E. coli*) detected in urine culture in a tertiary hospital where an antibiotic stewardship program is implemented on some of the restricted antibiotics. Time-series correlating the antibiotic consumption and prevalence of urinary ESBL-producing *E. coli* were hypothesized. Finally, time-dependent models of the effects of antibiotic consumption on the prevalence of urinary ESBL-producing *E. coli* were statistically proposed.

## Results

A total of 6603 urine cultures in which *E. coli* was identified were collected from 4395 patients who visited the emergency department or in-patient unit between 1 January 2012 and 30 June 2019. Duplicative isolates obtained from urine cultures that were conducted within a span of 30 consecutive days were regarded as one result. Among 4395 patients, 81.7% (n = 3589) were female. The average age of females and males was 67.6 ± 16.1 and 68.4 ± 14.4, respectively. We defined cases as community-onset cases when urine culture was collected in 48 h of admission and as hospital-acquired cases when the collection time exceeded 48 h. Among 5427 isolates, 64.3% (n = 3489) were considered as community-onset, and the average time difference between specimen collection and admission for these cases was 6.1 ± 12.5 h. For hospital-acquired isolates*,* the average time difference was 561.2 ± 1497.1 h. Moreover, among hospital-acquired isolates, 42.9% (n = 831) were identified as ESBL-producing *E. coli*, while 29.1% (n = 1014) of community-onset isolates were ESBL-producing *E. coli*.

**Figure 1:**
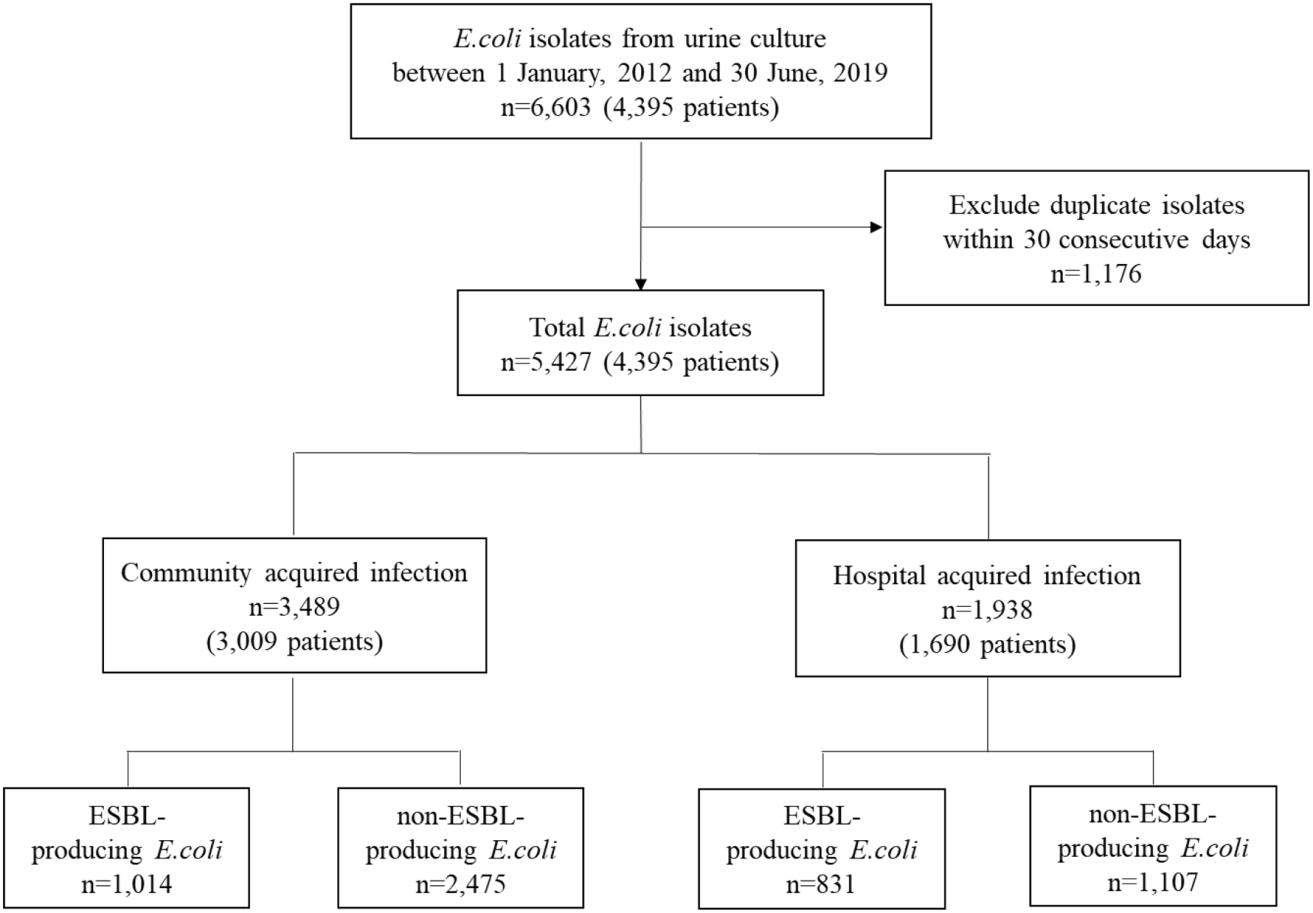
Flow diagram depicting the study population.

To establish the relationship between antibiotic consumption volume and the prevalence of hospital-acquired ESBL-producing *E. coli* isolates, trend and time-series analyses using the ARIMA model were conducted as described below (Fig. 1).

### ESBL-producing *E. coli* proportion and antibiotic usage trend

**Figure 2:**
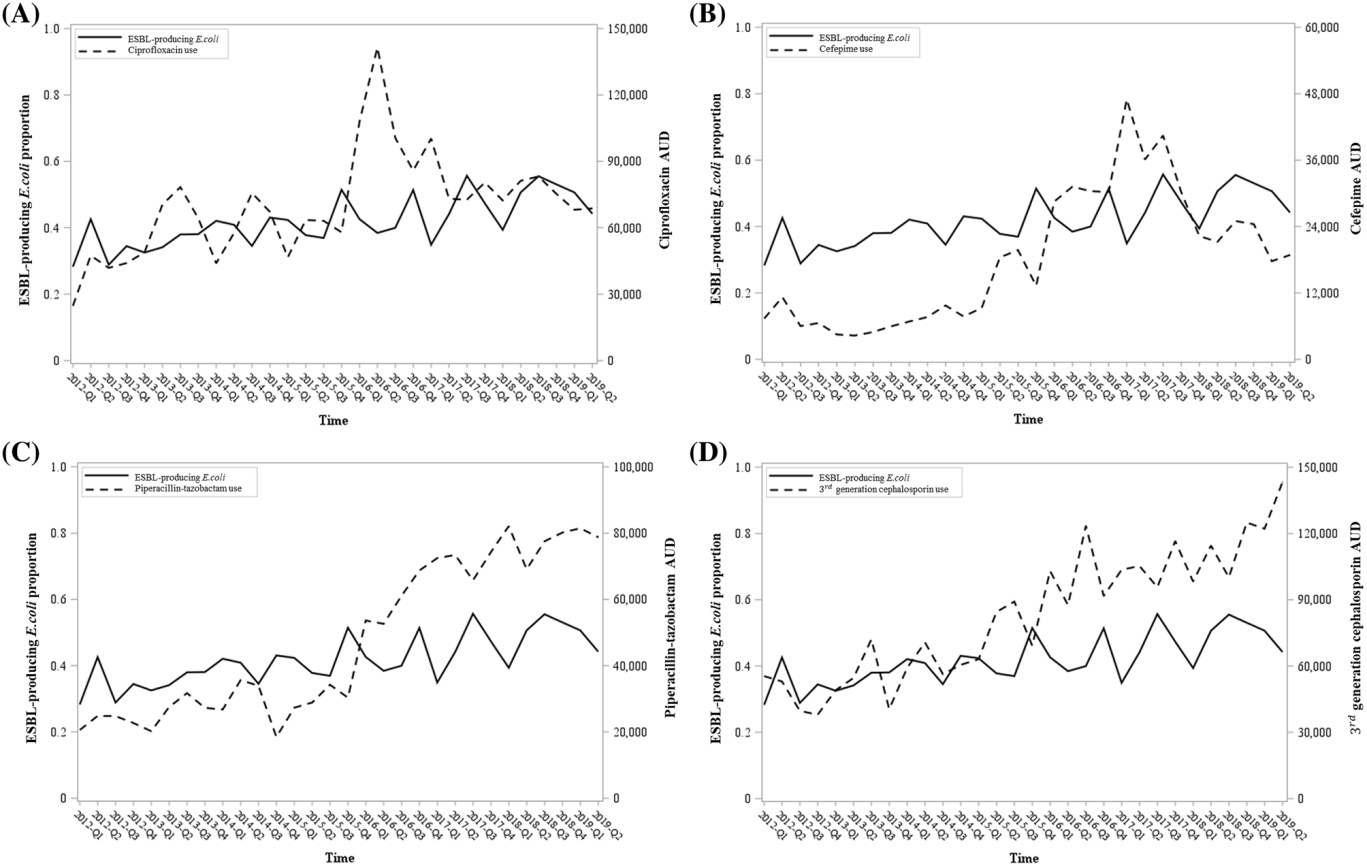
Time-series plot of ESBL-producing *E. coli* proportion and AUD from 1 January 2012 to 30 June 2019. AUD of (**A**) ciprofloxacin, (**B**) cefepime, (**C**) piperacillin-tazobactam and (**D**) third-generation cephalosporin.

The proportion of ESBL-producing *E.* coli increased over time ($$\upbeta =0.0062, P<0.0001)$$. Similarly, antimicrobial use density (AUD) of ciprofloxacin $$\left(\upbeta =1383.6622, P=0.0026\right),$$ cefepime $$\left(\upbeta =961.8701, P<0.0001\right),$$ piperacillin-tazobactam $$\left(\upbeta =2457.3924, P<0.0001\right),$$ and third-generation cephalosporine $$(\upbeta =$$ 3035.1029, $$P<0.0001)$$ consumption showed an increasing trend over time (Fig. 2).

### Correlation between ESBL-producing *E. coli* proportion and antibiotic usage

We identified a correlation between the proportion of ESBL-producing *E.* *coli* and antibiotic usage. The proportion of ESBL-producing *E. coli* was associated with ciprofloxacin usage two quarters earlier. Conversely, there was no correlation between the proportion of ESBL-producing *E.* *coli* and the usage of cefepime, piperacillin-tazobactam, and/or third-generation cephalosporin. Therefore, we used ciprofloxacin usage to build a transfer function model (Fig. S1).

### Ciprofloxacin usage and ESBL-producing *E. coli* proportion

The transfer function model was used to elucidate the relationship between ESBL-producing *E. coli* proportion and ciprofloxacin usage. Table 1 presents the transfer function model for ESBL-producing *E. coli* and ciprofloxacin AUD. The results show that an increase by 1 unit of ciprofloxacin usage is related to an increase in the proportion of ESBL-producing *E.* *coli* two quarters later by 0.0000024, and the proportion of ESBL-producing *E.* *coli* in the previous quarter was related to proportion of ESBL-producing *E. coli* in the present quarter. The transfer function model for the ESBL-producing *E. coli* proportion ($${Y}_{t})$$ and ciprofloxacin usage ($${X}_{t}$$) according to the Table 1 can be expressed as follows:

$$Y_{t} = 0.0000024X_{t - 2} + \frac{{a_{t} }}{1 - 0.9721B}$$

where $a_{t}$ denotes white noise and $B$ denotes back shift.

**Table 1:** ESBL-producing *E. coli* proportion and ciprofloxacin usage parameter estimates based on the transfer function model.

Parameter	Estimate	SE	*t* value	*p* value	Lag	Shift
AR1,1	0.9721	0.0361	26.91	< 0.0001	1	0
NUM1	0.0000024	0.0000006	4	< 0.0001	0	2

The autoregressive parameter was labelled as AR1,1: representing past value of ESBL-producing *E. coli* proportion. The numerator term was labelled as NUM1: ciprofloxacin usage. SE, standard error.

**Figure 3:**
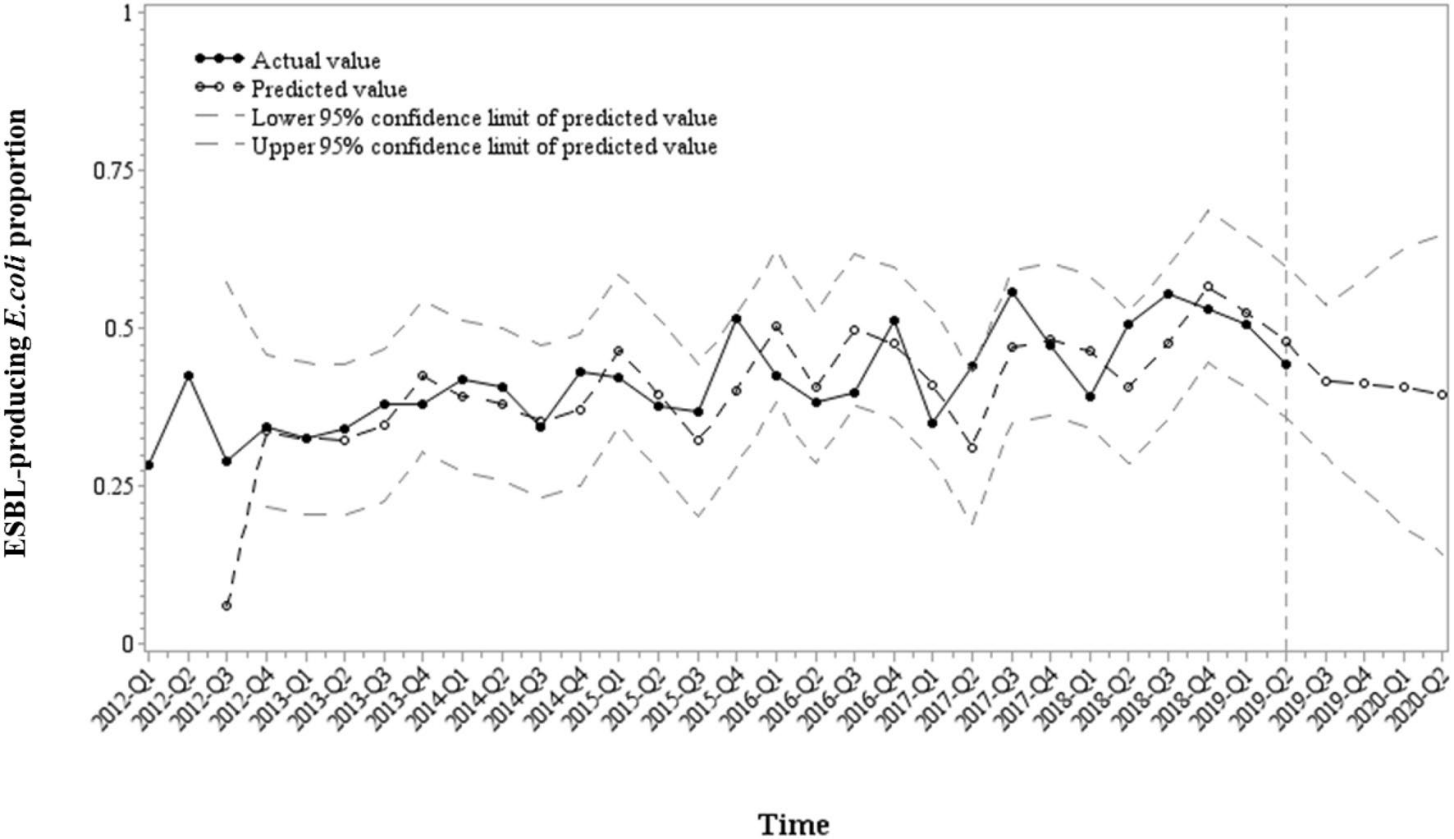
Predicted and observed ESBL-producing *E. coli* proportion upon ciprofloxacin usage.

The residual plots of the autocorrelation function (ACF) and partial autocorrelation function (PACF) showed that the assumptions of the transfer function model are acceptable, implying that the residuals correspond to the white noise (Fig. S2). The Akaike information criterion (AIC) and Schwarz–Bayesian information criterion (SBC) were − 71.9579 and − 69.2935, respectively, and the mean absolute percentage error (MAPE) value was 13.4336. The ESBL-producing *E. coli* proportion predictions for the next four quarters were 0.4182, 0.4124, 0.4065, and 0.3970 (Fig. 3).

## Discussion

The main accomplishments of the present study are as follows: (1) development of a unique method to process text-wise EHR data into standardized data made it possible to set up a model of large sets of data in a time-series; and (2) this time-dependent model demonstrated the volume of ciprofloxacin consumption in the six months prior to the prediction, predicts the proportion of ESBL-producing *E.* *coli*.

To elucidate the association between antibiotic consumption and related antimicrobial resistance, in the present study, a new database of antibiotic orders and bacterial culture reports, which were extracted and transformed from EHRs between 2012 and 2019, was established. Explorative data analysis revealed that the quarterly proportion of ESBL-producing *E. coli* in urine culture steadily increased over time. AUD of antibiotics of interest also showed an increasing trend over time. Among them, AUD of third-generation cephalosporin increased most exponentially with time and ranked the highest among all tested antibiotics. This in-hospital data correlated with the national reimbursement data demonstrating the total antibiotic consumption nationally, which also showed a significant increase in the trends of third-generation cephalosporin consumption in in-hospital settings^12^.

Time-dependent model of our data predicted that the detection of ESBL-producing *E. coli* in urine culture increased with the consumption of ciprofloxacin six months earlier than the present stipulated time. A previous study that was carried out in our center 12 years ago also showed that the proportion of ciprofloxacin-resistant *E. coli* was associated with consumption level in the 2 months prior to the measurement^8^. Although many ESBL-producing *E. coli* were ciprofloxacin resistant, their resistance remained in steady manner throughout the study period, while ESBL-producing *E. coli* proportion trend was increasing (Fig. S3). The present study revealed that the consumption of certain antibiotics increased not only its specific antibiotic resistance but also the expression of ESBL phenotype, further widening the spectrum of antimicrobial resistance. These results suggest that the level of in-hospital antibiotic usage is directly associated with an increase in antimicrobial resistance in nosocomial settings. Conversely, the restriction of using specific antibiotics may lead to a decrease in resistance to pathogens, which can be successfully implemented with antibiotic stewardship programs. Currently, ciprofloxacin in our center is not included in the in-hospital antibiotic stewardship program and was freely prescribed by physicians without any restrictions or recommendations. However, an indiscreet usage of antibiotics should be avoided, restriction on antibiotic usage should also be implemented cautiously to avoid adverse effects, such as increased mortality or longer duration of hospital stay. Despite reducing antibiotic consumption, antimicrobial resistance might still persist due to its relatively low fitness cost^13^. Notably, Lozano et al.^14^ identified the minimum threshold of fluoroquinolone usage in lowering ESBL-producing *E. coli* below 100 DDDs per 1000 occupied bed days using a nonlinear time-series analysis, which our center far exceeds.

Other studies have shown a strong relationship between the usage of third-generation cephalosporin and expression of ESBL phenotype^3,15,16^. Tacconelli et al.^3^ reported that cephalosporin monotherapy ranked first among all antibiotics in promoting colonization of ESBL gram-negative bacteria in rectal swabs through analyzing the patient-level data with machine learning methods. However, in our study, this correlation was not evident. This was an unexpected result considering that third-generation cephalosporin was the most frequently used antibiotic in hospitals. One way to explain this result is that third-generation cephalosporin has been managed by antimicrobial stewardship program since 2002 in our center. It was prescribed only under the authorization of antibiotic use program by infectious disease department specialists to minimize its unnecessary usage and misuse. This situation in the center might have led to the above results, implying that antimicrobial stewardship program may exert antibiotic selective pressure. Although there are controversial data on the effect of antimicrobial stewardship program on antibiotic resistance^9^, similar results have been reported in a recent study conducted by Hwang et al.^17^ using interrupted time-series analysis, which showed a decrease in the antimicrobial resistance rate of *Staphylococcus aureus* and *Pseudomonas aeruginosa* when antimicrobial stewardship intervention was implemented. Therefore, using broader classes of restricted antibiotics, such as fluoroquinolones, in antimicrobial stewardship programs should be considered to restrict ESBL-producing *E. coli*.

Regarding cefepime, it was difficult to obtain a significant relationship between AUD and the proportion of ESBL-producing *E. coli*. The consumption of piperacillin-tazobactam and proportion of ESBL-producing *E. coli* were correlated with a time interval of 33 months (lag of 11), which was very difficult to interpret clinically. Cefepime usage did not show a significant correlation with ESBL-producing *E. coli.* Cefepime and piperacillin-tazobactam antibiotics were already included in the hospital antibiotic stewardship program and were regulated along with third-generation cephalosporin. In particular, our center strongly recommended the usage of piperacillin-tazobactam and cefepime as selective antibiotics for ESBL-producing urinary tract infections, rather than carbapenem, based on the carbapenem-sparing strategy. This means that the level of piperacillin-tazobactam and cefepime usage in hospitals is dynamically influenced by the current proportion of ESBL-producing *E. coli*. This relationship might have acted as a confounding factor in the association of the antibiotics with ESBL-producing *E. coli*.

This study has some limitations. First, the time-series transfer function model reflects the data collected at different time points. Therefore, other clinically relevant characteristics were not included in the model, such as sex, co-morbidity, and invasive procedure such as indwelling catheter. Further investigation on predicting patient-level ESBL-producing organism proportions should be conducted using other models. Second, expansion of ESBL-producing *E. coli* due to nosocomial transmission or in-hospital outbreaks instead of antibiotic selective pressure was not taken into consideration. This clinical situation may have led to misinterpretation. Third, the transfer function model built using ciprofloxacin showed a slightly high MAPE value of 13.4336, indicating that the forecast can be off by 13.4336%. However, by using longer time-series data and taking into consideration other risk factors, forecasting measurement error can be reduced^18^. Fourth, the size of the hospital might be a potential confounding factor. The effect of antimicrobial consumption on the prevalence of ESBL-producing *E. coli* might be different in small- or large-sized hospitals. Therefore, a multi-center study including both small and large hospitals should to be performed in the future.

Despite these limitations, the present study demonstrates the relationship between antibiotic consumption and the prevalence of antimicrobial resistance. Efforts to reduce inappropriate antibiotic usage, especially of fluoroquinolones in clinical practice, should be made in hospitals through antimicrobial stewardship programs. Moreover, the development of algorithms for extracting the text-wise data from electronic health records provides a much easier approach for the use of antimicrobial data from other hospitals in addition to that from our center. Further research on other data sets may provide insights into additional relationships between AUD and antimicrobial resistance by using a transfer function model as well as other methods, such as other mathematical models or machine learning.

## Methods

### Data approval

The study protocol was approved by the Institutional Review Board of Korea University Anam Hospital (IRB No. 2019AN0228). Written informed consent was waived by the Institutional Review Board of Korea University Anam Hospital because of the retrospective study design with minimal risk to participants. The study complied with the principles of the Declaration of Helsinki.

### Bacterial culture report data processing

The bacterial culture reports from 1 January 2012 to 30 June 2019 were extracted from the electronic health record (EHR) database. Since the bacterial culture reports were in a semi-standardized free-text format, detailed standardized data were further extracted using text processing techniques, including regular expressions. The names of the bacterial organisms and antimicrobials were standardized.

### Microbiological evaluation

*Escherichia coli* in the urine culture specimen was identified using the VITEK II (bioMèrieux, Hazelwood, MO, USA) system and matrix-assisted laser desorption/ionization-time of flight (MALDI-TOF) mass spectrometry (Bruker, Coventry, UK). Antibiotic susceptibility testing was performed using the VITEK II (bioMèrieux, Hazelwood, MO, USA) system or MicroScan WalkAway 96 plus (Siemens Healthcare Diagnostics Inc., CA, USA) system following the Clinical and Laboratory Standards Institute (CLSI) guidelines^19^. Phenotypic screening for ESBL production was performed using the VITEK II (bioMèrieux, Hazelwood, MO, USA) system.

### Antimicrobial consumption data

Pharmacological data were determined according to the Anatomical Therapeutic Chemical (ATC) classification with the defined daily dose (DDD) as a unit of measure, as recommended by the WHO Collaborating Centre for Drug Statistics Methodology^20^. DDD is defined as the assumed average maintenance dose per day for a drug used for its main indication in adults. Data of five classes of antibiotics of interest (ciprofloxacin, third-generation cephalosporin including ceftriaxone, cefotaxime, and ceftazidime, cefepime, trimethoprim-sulfamethoxazole, and piperacillin-tazobactam) were extracted from EHR in grams and international unit form and converted into DDD. AUD was used to estimate the quarterly usage of antibiotics. AUD was calculated as follows:

$${\text{AUD}} = \left[ {\frac{{{\text{total}}\,{\text{antibiotic}}\,{\text{consumption}}\,{\text{amount}}\left( {\text{mg}} \right)}}{{{\text{DDDs }}\left( {{\text{mg}}} \right)\times}{\text{patients}}\,{\text{per year}}\,}} \right] \times 1000\,$$

### Statistical analysis

Using linear regression, quarterly trends in the proportion of ESBL-producing *E. coli* and antibiotic usage were verified over time. We applied a transfer function model to investigate the association between antibiotic consumption and the proportion of ESBL-producing *E. coli*. The modeling was performed following three procedures: (1) building an adequate ARIMA model for two time-series, antibiotic use, and ESBL-producing *E. coli* proportion; (2) identifying the cross-correlation between two time-series to determine the systematic part of the transfer function; and (3) diagnostic checking of the chosen model: autocorrelation function (ACF) and partial correlation function (PACF) of residuals were identified to determine whether they were contributing to the white noise. The AIC and SBC were computed for evaluating goodness of fit and mean absolute percentage error (MAPE) for the measurement of forecast error.

$${\text{MAPE}} = \frac{1}{N}\mathop \sum \limits_{t}^{N} \frac{{\left| {Y_{t} - \widehat{{Y_{t} }} } \right|}}{{Y_{t} }} \times 100.$$

where ${Y}_{t}$ denotes the observed ESBL-producing *E. coli* proportion at month t and $\widehat{{Y}_{t}}$ denotes the forecasted ESBL-producing *E. coli* proportion at month t. All statistical analyses were performed using SAS 9.4 (SAS Institute Inc., Cary, NC, USA) program.

## Supplementary Information

Supplementary Information.

## Data Availability

The datasets used and analyzed during the present study can be available form the corresponding author, Jang Wook Sohn, upon reasonable request.
