# Supplementary material for: Impact of antibiotic usage on extended-spectrum β-lactamase producing Escherichia coli prevalence
Source: Sci Rep. 2021 Jun 22;11:13024. doi: 10.1038/s41598-021-91332-x (PMC8219833; doi:10.1038/s41598-021-91332-x)

Impact of antibiotic usage on extended-spectrum ß-lactamase producing *Escherichia coli* prevalence

Jeong Yeon Kim^#1^, Yun Jin Yum^#2^, Hyung Joon Joo^3,4^, Hyonggin An^2,3^, Young Kyung Yoon^1^, Jong Hun Kim^#5*^, Jang Wook Sohn^#1,3*^

^1^Division of Infectious Diseases, Department of Internal Medicine, Korea University College of Medicine, Seoul, Republic of Korea

^2^Department of Biostatistics, Korea University College of Medicine, Seoul, Republic of Korea

^3^Korea University Research Institute for Medical Bigdata Science, Korea University, Seoul, Republic of Korea

^4^Department of Cardiology, Cardiovascular Center, Korea University College of Medicine, Seoul, Republic of Korea

^5^Division of Infectious Diseases, Department of Internal Medicine, CHA Bundang Medical Center, CHA University, Seongnam, Republic of Korea

# Contributed equally

^*^Corresponding authors

Jong Hun Kim (smonti1976@hotmail.com) & Jang Wook Sohn (jwsohn@korea.ac.kr)

**Figure S1.** Cross correlations function of ESBL-producing *E.coli* proportion and AUD. AUD of (A) ciprofloxacin (B) cefepime (C) piperacillin-tazobactam (D) third generation cephalosporin. CCF, cross correlation function.


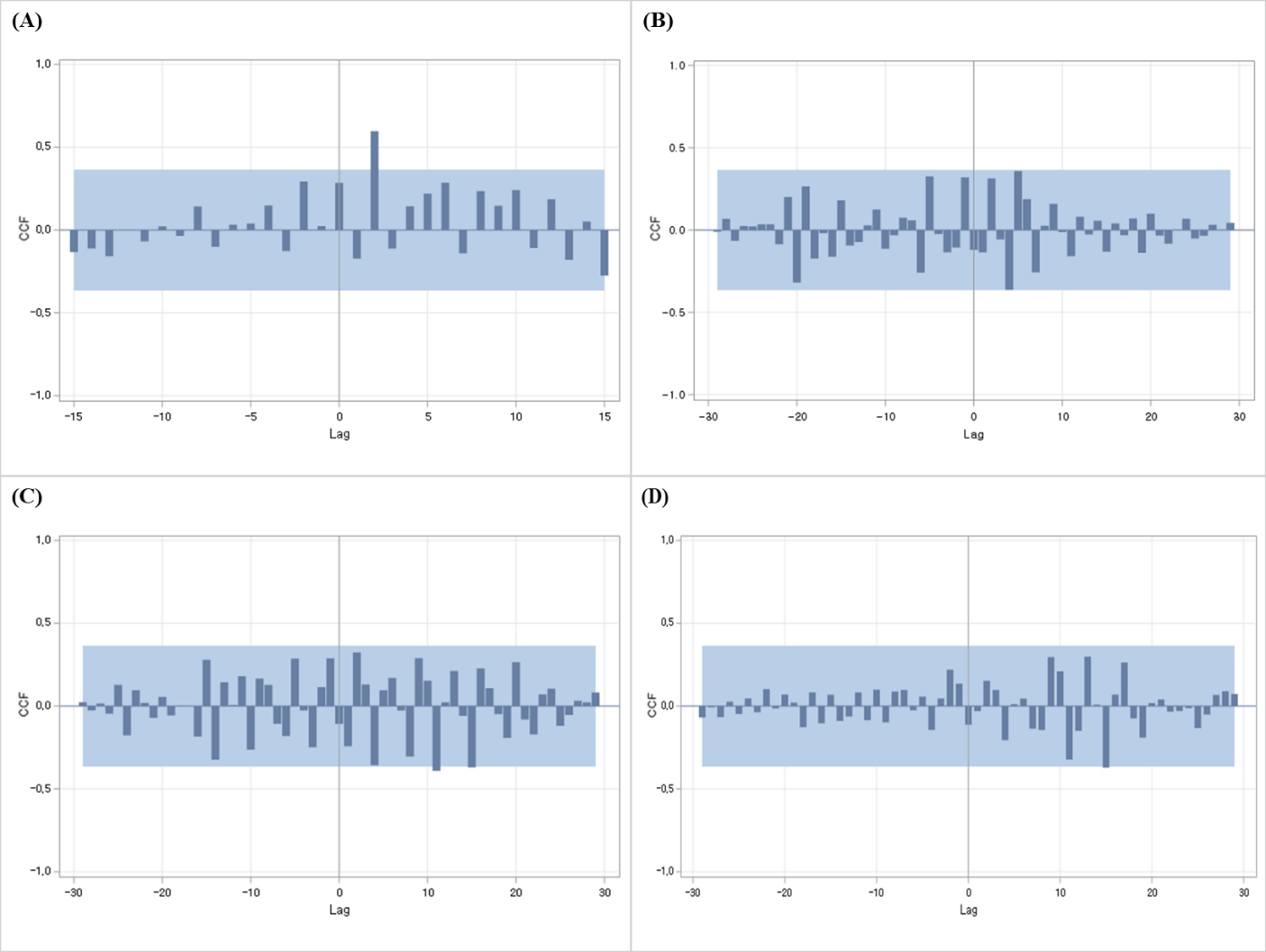


Figure S2. Autocorrelation function and partial autocorrelation function of the ESBL-producing *E.coli* proportion and ciprofloxacin consumption model. (A) ACF (B) PACF. ACF, autocorrelation function; PACF, partial autocorrelation function.


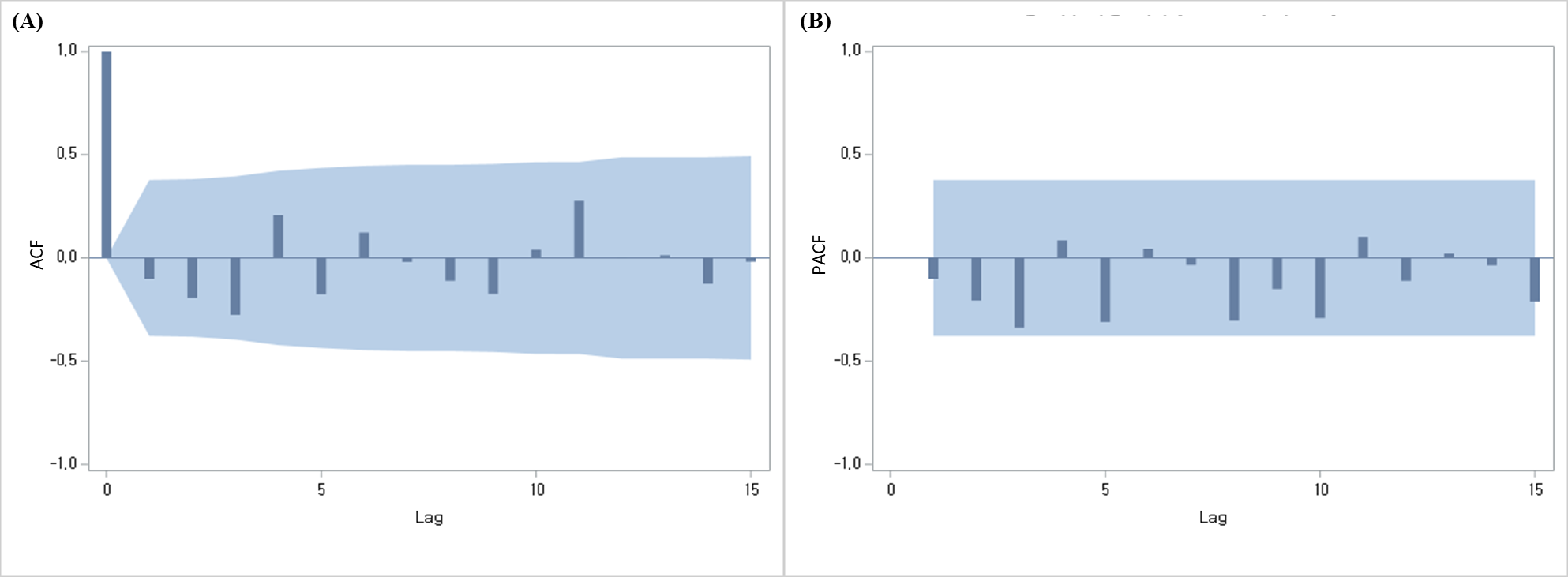


Figure S3. Ciprofloxacin resistance proportion of ESBL-producing E. coli during study period.


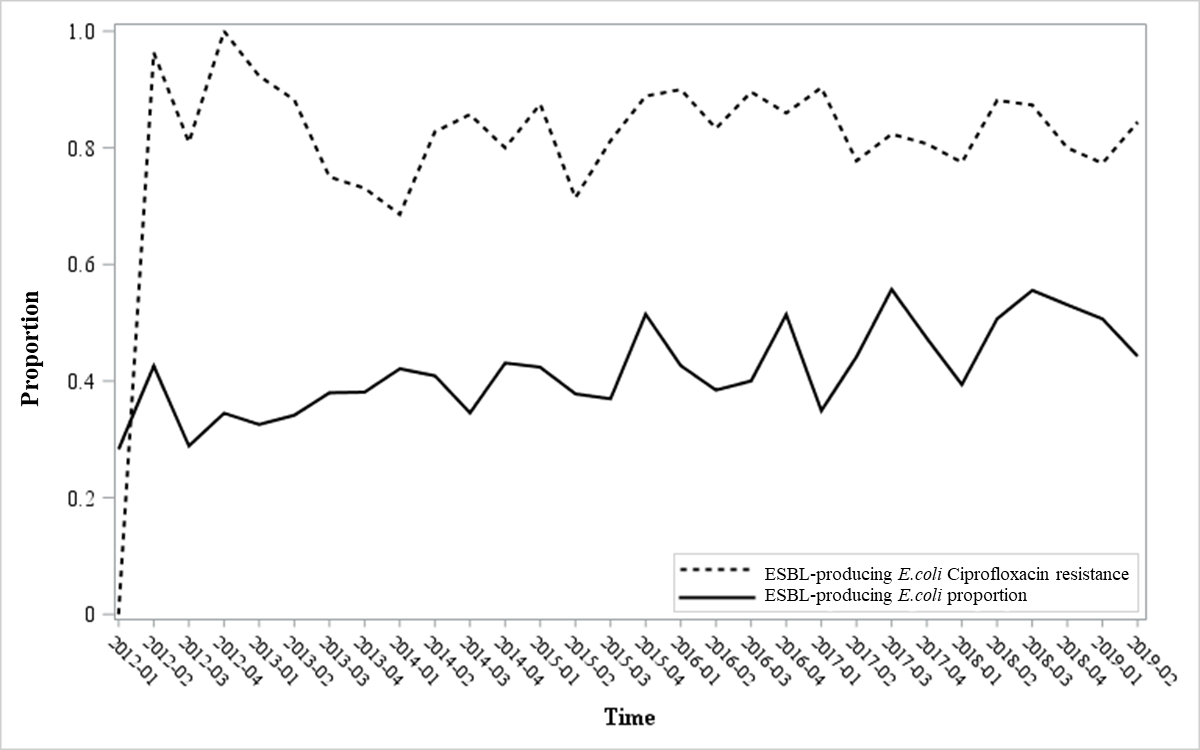

Supplement: Supplementary file 1 — Supplementary Information. [file 41598_2021_91332_MOESM1_ESM.docx]
